# p21^WAF1/CIP1^ Expression is Differentially Regulated by Metformin and Rapamycin

**DOI:** 10.1155/2014/327640

**Published:** 2014-03-25

**Authors:** Zoltan Molnar, Ann B. Millward, Wai Tse, Andrew G. Demaine

**Affiliations:** Renal Unit and Diabetes Clinical Research Unit, Derriford Hospital, Plymouth, PL6 8DH, UK

## Abstract

The mammalian target of rapamycin (mTOR) pathway plays an important role in the development of diabetic nephropathy and other age-related diseases. One of the features of DN is the elevated expression of p21^WAF1/CIP1^. However, the importance of the mTOR signalling pathway in p21 regulation is poorly understood. Here we investigated the effect of metformin and rapamycin on mTOR-related phenotypes in cell lines of epithelial origin. This study reports that metformin inhibits high glucose-induced p21 expression. High glucose opposed metformin in regulating cell size, proliferation, and protein synthesis. These effects were associated with reduced AMPK activation, affecting downstream mTOR signalling. However, the inhibition of the mTOR pathway by rapamycin did not have a negative effect on p21 expression, suggesting that metformin regulates p21 upstream of mTOR. These findings provide support for the hypothesis that AMPK activation may regulate p21 expression, which may have implications for diabetic nephropathy and other age-related pathologies.

## 1. Introduction

There has been a dramatic increase in the prevalence of diabetes mellitus in recent years [1]. The chronic effects of diabetes may manifest in macro- and microvascular complications that are the major causes of morbidity and mortality in patients with diabetes. Diabetic nephropathy (DN), one of the microvascular complications, is a leading cause of death from kidney failure [2, 3]. Apart from haemodynamic factors, hyperglycaemia has been shown to be an underlying cause of pathogenesis in DN. The damaging effects of hyperglycaemia have been partly attributed to increased cellular glucose uptake in cells that are not protected from high ambient glucose levels. Early cellular changes in the development of DN include hyperplasia and hypertrophy [4].

Several investigators have associated the expression of Cip/Kip cyclin-dependent kinase (CDK) inhibitors, p21 and p27, with glomerular hypertrophy [5–7]. It has been proposed that p21 and p27 may be involved in hypertrophy independently of their cell cycle regulatory properties (Monkawa 2002). Furthermore, the induction of p21 and p27 is also required for senescent arrest, a molecular signature of hypertrophic changes in the early stages of the development of diabetic kidney disease [8]. The fact that p21 null mice do not develop glomerular hypertrophy supports the importance of p21 in DN [9].

The activation of the mammalian target of rapamycin (mTOR), a serine/threonine kinase, plays a pivotal role in the pathologic forms of hypertrophy in the kidneys [10–12]. mTOR forms two complexes with distinct functional and physical properties. These complexes have two different scaffolding proteins, raptor and rictor. By interacting with distinct downstream targets, these scaffolding proteins connect mTOR to different signalling pathways, resulting in discrete functional roles [13].

The raptor-mTOR protein complex is rapamycin sensitive; it integrates extracellular and intracellular signals originating from growth factors, hormones, and nutrients. This complex plays a key role in regulating the cellular response to nutrients by phosphorylating the downstream target proteins, P70S6 Kinase1 (S6K) and initiation factor 4E [14]. Studies on skeletal muscle cells have shown that, through a negative feedback mechanism, the activation of the mTOR pathway may lead to insulin resistance [15]. Furthermore chronic rapamycin treatment in rats induced the expression of hepatic gluconeogenic enzymes, which may adversely affect glucose levels in a diabetic state [12]. On the other hand, it has been shown by several investigators that the inhibition of the mTOR signalling pathway has a therapeutic potential for the treatment of DN [13, 16].

mTOR is also regulated by AMP-activated protein kinase (AMPK), a sensor of intracellular AMP levels [17]. Mammalian AMPK is a heteromeric complex consisting of one catalytic subunit *α* and regulatory *β*, *γ* subunits. Through a conformational change in the *γ* subunit, AMP facilitates the phosphorylation of Thr-172 on the *α* subunit by various upstream kinases, including Ca^2+^-calmodulin-dependent kinase *β*, TGF-*β*-activated kinase 1, and LKB1 serine/threonine kinase [18]. AMPK activation has been generally associated with inhibition of mTOR signalling. The antidiabetic drug metformin depletes cellular ATP levels by blocking mitochondrial respiratory complex I. In turn, the elevated AMP levels induce the activation of AMPK [19]. Nevertheless, it has been suggested that metformin may activate AMPK through an AMP-independent mechanism [20]. Although AMPK activation results in the inhibition of mTOR signalling, recent findings also suggest that metformin may abolish mTOR activation independently of AMPK [17]. Apart from its insulin-sensitising properties, metformin may have several beneficial effects in various clinical settings [21].

According to clinical studies in both type 1 and type 2 diabetes, longstanding hyperglycaemia is the primary cause of DN [11, 22]. Several studies have shown that excess glucose increases cell size in various cell types through the activation of Akt-mTOR signalling; however, the mechanism leading to glucose-induced mTOR activation has not been well defined. It has been suggested that the hypertrophic changes induced by hyperglycaemia may be the consequence of mTOR activation through autocrine TGF-*β* signalling [23]. In addition, mTOR activity has also been associated with increased expression of the glucose transporter 1 (GLUT1) in mesangial cells [24]. However, saturation of glucose uptake in mesangial cells has been reported to occur at 30 mM, indicating that hyperglycaemia can induce mTOR in the absence of increased GLUT1 expression [25].

The aim of this study was to compare the inhibitory effects of rapamycin and metformin on proliferation and cell growth in the context of high glucose-induced AMPK/mTOR signalling. We have observed differential effects of metformin and rapamycin in several AMPK/mTOR-related aspects with relevance to dysregulated cell growth and cell cycling in DN.

## 2. Materials and Methods

### 2.1. Cell Culture, Treatments, and Transfection

Human embryonic kidney (HEK293) cell line was maintained in Minimum Essential Media (Invitrogen) supplemented with 10% FBS, 1% Pen/Strep (Invitrogen), and 1% MEM nonessential amino acid solution. The cells were cultured at 37°C in a 95% air/5% CO_2_ environment and passaged every 3-4 days at subconfluence.

Conditionally immortalised human podocyte cells (courtesy of Professor Saleem, Bristol) were cultured in RPMI 1640 medium containing 11 mM D-glucose, 10% foetal bovine serum 1% Penicillin/Streptomycin. Briefly, the cell line was generated by isolating podocyte cells from a normal human kidney specimen and transfected with a temperature sensitive simian virus-40 large T-antigen. The cells proliferate at a permissive 33°C, then culturing the cells at 37°C switches off T-antigen expression, allowing the cells to assume a native phenotype. In the experiments, these cells were used under growth-permissive conditions.

The cells were cultured with different concentrations of D-glucose (Sigma-Aldrich). Where appropriate, mannitol (Sigma-Aldrich) was used to control osmotic effects. Rapamycin stock solutions were prepared in 95% ethanol. The final concentration of ethanol was maintained below 0.1%. Metformin (Sigma-Aldrich) stock solution was prepared in phosphate buffered saline (PBS). Stock solutions were filter sterilized. Vehicle controls were included in each experiment and exerted no effect on cell viability.

Eight pGIPZ lentiviral shRNA constructs against human AMP-activated, alpha2 catalytic subunit (PRKAA2), and a nonsilencing construct were purchased from Open Biosystems. pGIPZ plasmids were stored in bacterial cultures of* E. coli* (Prime Plus) in LB Lennox (5 g NaCl/L) with 8% glycerol, 100 *μ*g/mL carbenicillin, and 25 *μ*g/mL zeocin. Isolation of plasmid DNA was done by Plasmid Midi Kit (Qiagen) according to the protocol supplied by the manufacturer. Stable cell lines were generated by transfecting HEK293 cells with 2 *μ*g/mL plasmid DNA in 24 well plates using Arrest-in Transfection Reagent (Open Biosystems). Selection for stably transfected cells was done in medium supplied with 8 *μ*g/mL puromycin.

### 2.2. Proliferation Assay

The cell suspension was loaded in 96 well microtitre plates at 1 × 10^4^/mL density and allowed to grow until 50% confluence when the cells were exposed to experimental conditions. Cell proliferation was determined using the Cell Titre96 AQ_ueous_ Non-Radioactive Cell Proliferation Assay (Promega). The proliferation assay is an MTS-based method that spectrophotometrically measures the conversion of tetrazolium salt into a formazan product. The absorbance of this product was measured at 490 nm by spectrophotometry. The quantity of this product is directly proportional to the number of living cells in culture.

### 2.3. Flow Cytometry

Flow cytometry was performed to analyse cell cycle distribution and cell size in HEK293 cells. After treatment, the cells were trypsinised and washed with PBS by centrifuging them twice at 200 g for 5 minutes (min). The cells for cell cycle analysis were fixed in cold saline GM and 90% ethanol (1 : 3 ratio) and stored at –80°C. Before analysis, the cells were centrifuged at 500 g for 5 min, resuspended in FACS buffer (PBS, 2% FCS, 10 mM sodium azide), and treated with 100 *μ*g/mL RNase A (Sigma). DNA was stained with 50 *μ*g/mL propidium iodide (Sigma) for 1 hour (h) at room temperature (RT), and the percentage of 1 × 10^4^ cells in the G1/G0, S, and G2/M phases cells was determined. The cells for cell size analysis were resuspended in FACS buffer and relative forward side scatter determined on a Becman-Coulterton Epics XL.MCL flow cytometer running EXPO32 ADC software (10,000 events). In order to investigate the viability of the cells, propidium iodide staining was used. Floating and adherent cells were harvested by trypsinisation, pelleted by centrifugation at 200 g for five minutes, and then washed twice with FACS buffer. The pellet was resuspended in FACS buffer with 20 *μ*g/mL propidium iodide, incubated on ice for 10 minutes, and analysed for dye inclusion on a BD Accuri C6 flow cytometer.

### 2.4. Protein Extraction and Western Blotting

Cell proteins from at least two independent experiments were extracted by addition of lysis buffer containing 20 mM Tris-HCl (pH 7.4), 150 mM NaCl, 1 mM EDTA, 1% TX100, and 1 tablet/10 mL PhosSTOP Phosphatase Inhibitor (Roche). The suspension was centrifuged at 14000 ×g and the supernatant containing cellular protein was collected. For western blotting, a 12.5% sodium dodecyl sulphate polyacrylamide gel was run under standard conditions, loading 25 *μ*g of total protein in each lane. The gel was placed in transfer buffer and set up for transfer onto a polyvinylidene fluoride membrane at 250 mA overnight. The membrane was rinsed in Tris-buffered saline immersed in blocking buffer (2% BSA) for 1 h then incubated with primary antibodies (P-mTOR Ser^2448^, P-AMPK*α* Thr^172^, P-S6K Thr^389^, p21, 1 : 1000, and cell signalling) overnight at 4°C. After rinsing in wash buffer, the membrane was incubated with horseradish peroxidase-conjugated secondary antibody (cell signalling) for 1 h at 1 : 5000 dilution at RT. An enhanced chemiluminescence kit (Amersham) was used for detection of the bands. In order to control protein loading, total-mTOR, and *β*-actin antibodies were used (1 : 1000, cell signalling).

### 2.5. Immunocytochemistry

HEK293 cells were cultured on glass slides to reach 60% confluence. After exposure to 8 mM metformin for 24 h, the cells were fixed with 4% paraformaldehyde in PBS for 15 min at RT. The slides were washed in PBS, permeabilized, and blocked with 0.2% Triton X-100, 10% FCS, 125 mM L-lysine, and 10% sodium azide for 30 min. The slides were washed and incubated with the primary antibody at 1 : 400 dilution (p21, cell signalling) overnight. The cells were rinsed and subsequently incubated with Alexa Fluor 568 (Invitrogen) goat anti-mouse secondary antibody at 1 : 500 and DAPI at 1 : 1000 dilutions for 1 h at RT. The slides were analysed by fluorescent microscopy (Nikon eclipse 80i microscope and a Nikon digital colour camera). Controls included the omission of primary antibody.

### 2.6. Total Protein/Cell Number Ratio

Total protein/cell number ratio was used to determine whether the alteration of cell growth in response to high glucose treatment was accompanied by cell hypertrophy. For the experiments, HEK293 cells were cultured in Minimal Essential Medium. At the end of the treatment period, the cells were trypsinised and washed twice with PBS and counted in a haemocytometer chamber. The cells then were lysed to measure the total protein content by the BCA protein assay (Thermo Scientific). The total protein/cell number ratio expressed as *μ*g/10^5^ cells was used as a hypertrophy index.

### 2.7. Data Analysis

All data analyses were performed with IBM SPSS 19. Data are expressed as means ± SEM. Normality of distribution was verified by Shapiro-Wilk test and homogeneity of variance by Levene's test. Unless otherwise indicated, statistical significance of differences was calculated by *t*-test or one-way ANOVA. Comparison among groups was conducted with Tukey's posthoc test. Differences were considered significant at *P* < 0.05.

## 3. Results

### 3.1. Dose-Response Studies of the Effects of Metformin and Rapamycin on HEK293 Cell Proliferation

**Figure 1:**
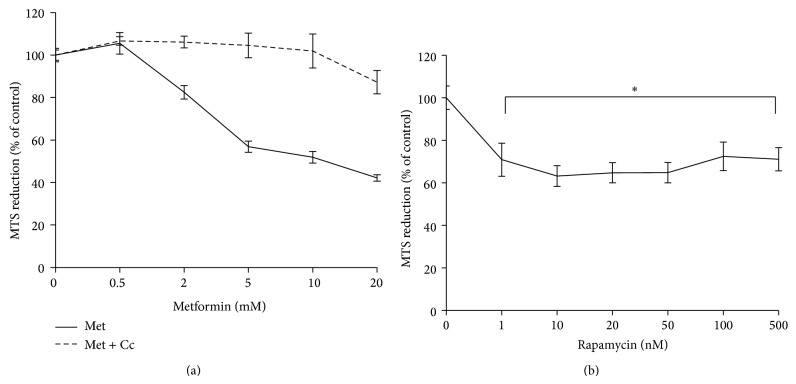
Inhibition of HEK293 cell proliferation by metformin (a) and rapamycin (b). 5000 cells per well were seeded in 96-well plates in MEM, cultured overnight and then exposed to increasing concentrations of metformin (Met) and rapamycin for 24 h. In combination with metformin treatments, 20 *μ*M of compound C (Cc) was used. Cell proliferation was measured by the MTS cell proliferation assay. Composite results expressed as mean ± SEM of three independent experiments. (a) Mixed ANOVA: within subject effects of metformin concentration (*F* (5,85) = 52.48  *P* < 0.001), between subject effects of combination treatments with compound C *F* (1,17) = 52.15  *P* < 0.001. (b) ^*^ *P* < 0.05, compared with 100%.

In order to characterise the effects of metformin and rapamycin on HEK293 cell proliferation, a dose-response study was carried out, using the MTS assay. In other studies the effects of metformin and rapamycin have already been compared by using the Alamar blue metabolic assay [26]. The investigators have found a concentration-dependent difference between the effects of metformin and rapamycin on proliferation. In order to test whether their result could be reproduced by using the MTS assay, the two drugs were used in the same concentration ranges [26]. The results show that over a 24 h period, metformin and rapamycin inhibited cell growth in HEK293 cells in a dose-dependent manner (Figure 1). However, increasing concentrations of metformin inhibited proliferation in a linear fashion, whereas increasing the concentration of rapamycin in the range of 10–500 nM did not cause a significant change in cell proliferation. Although Zakikhani et al. [26] used lower serum concentration and longer incubation time; the findings presented here are in line with their results, showing that the inhibitory effect of metformin on cell proliferation is concentration-dependent and, at higher concentrations, more pronounced than that of rapamycin. Furthermore, 20 *μ*M compound C (dorsomorphin), a research compound widely used for the inhibition of AMPK had an opposing effect on metformin-induced decrease in proliferation.

### 3.2. High Glucose-Treated HEK293 Cells Differentially Respond to Cell Cycle Inhibition by Metformin and Rapamycin

The purpose of this experiment was to investigate how cell cycling is affected by metformin and rapamycin treatments, and whether cell cycle changes are modulated by high glucose concentration. The effect of metformin on cell growth and proliferation has been investigated by various cell culture studies [17, 27, 28]. In these studies metformin was used at a concentration range of 10–20 mM. The use of these relatively high concentrations of metformin may be attributed to the low uptake of this drug by immortalised cell lines [21]. In the context of the inhibition of hepatic gluconeogenesis, the use of metformin in the millimolar range has been shown to be physiologically relevant [19]. The inhibitory effect of 8 mM metformin on cell cycle progression has been demonstrated in a cell culture study of breast cancer cells. In susceptible cells this concentration caused an approximately 50% reduction in cell number over a 24 h culture period [29]. As demonstrated in Figure 1, at 8 mM concentration, metformin caused an approximately 50% reduction in proliferation, suggesting that our findings may be comparable to earlier studies [29]. In this experiment metformin was used at 8 mM and its effect on cell cycling was compared to that of rapamycin. The effect of rapamycin on proliferation has also been studied in vascular smooth muscle cells. The inhibitory effect of rapamycin on proliferation has been shown in a range of concentrations (1–100 ng/mL). It has also been reported that rapamycin did not affect the viability of cultured cells in this concentration range [30]. In order to maximise its effect on cell cycling, in this experiment rapamycin was used at 100 nM.

In cell culture models of diabetes high glucose concentrations range from 25 to 30 mM [31]. It has also been shown by several investigators that after 24 to 72 h high glucose induces cellular changes implicated in the development of diabetic nephropathy, including increased protein synthesis, proliferation, and the expression of TGF-*β*. In these studies, up to 72 h culture period, high glucose did not have a significant effect on viability [32–35]. In order to study whether high glucose influences cell cycling, 30 mM D-glucose treatment was used for 72 h. In order to control the osmotic effects of high glucose, the normal culture media containing 5.5 mM D-glucose was supplemented with 24.5 mM mannitol. This sugar is widely used for similar research purposes [36–38].

**Figure 2:**
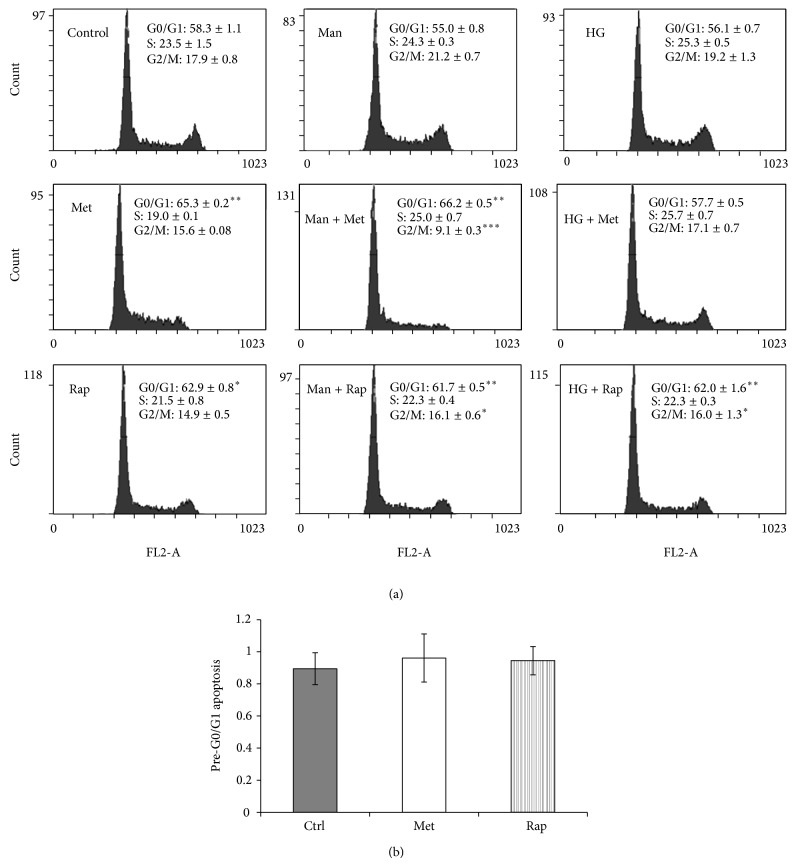
(a) High glucose inhibits metformin-induced cell cycle arrest in G0/G1 phase. HEK293 cells were cultured in 5.5 mM (Control), 5.5 mM D-glucose + 24.5 mM mannitol (Man), and 30 mM D-glucose (HG) for 3 days. For the last 24 hours, metformin 8 mM (Met) or rapamycin 100 nM (Rap) was added. Representative flow cytometry panels containing data expressed as mean per cent cell cycle distribution + SEM. ^*^ *P* < 0.05; ^**^ *P* < 0.01;  ^***^ *P* < 0.001, relative to control. (b) Percentage of cells in pre-G0/G1 apoptosis. Data expressed as mean ± SEM.

The results of cell cycle analysis show that both metformin and rapamycin increased the percentage of cells in the G0/G1 phase (Figure 2(a)). In comparison, the effect of metformin on cell cycle inhibition was higher than that of rapamycin. In the high glucose treated conditions, however, metformin-induced cell cycle arrest was abrogated. In contrast, high glucose did not have an effect on rapamycin-induced cell cycle arrest. The probability that osmotic effects played a role in the reversal of metformin-induced cell cycle arrest by high glucose is low, as mannitol at an equimolar concentration to high glucose, did not influence cell cycling in the same way. Nevertheless, mannitol treatment caused a significant decrease in the G2/M phase in the metformin-treated condition. This effect in the mannitol-only condition could not be observed. Since apoptosis can be induced through G0/G1 arrest, it was important to test whether cell cycle arrest mediated by metformin and rapamycin could have been associated with increased apoptosis [39]. To investigate this, the percentage of pre-G0/G1 cells was measured as described in the methods. The results indicate that neither metformin nor rapamycin caused a statistically significant increase in the percentage of pre-G0/G1 cells (Figure 2(b)).

#### 3.2.1. Cell Cycle Inhibition by Metformin Is AMPK-Dependent

**Figure 3:**
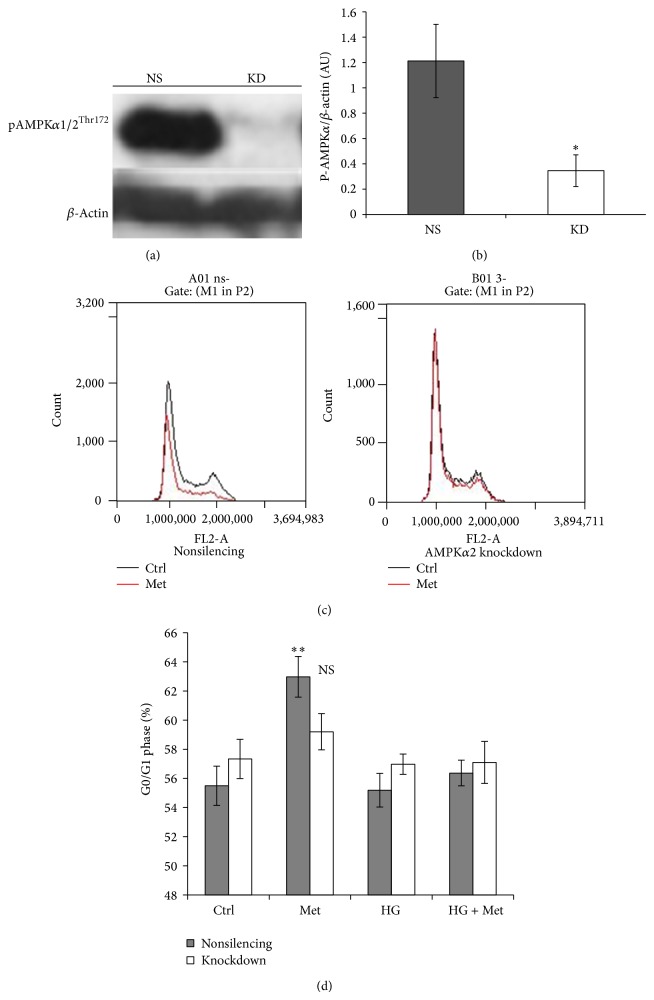
Metformin does not induce cell cycle arrest in AMPK*α*2-deficient cells. (a) Representative western blot image showing the effect of AMPK*α*2 knockdown, nonsilencing control (NS), knockdown (KD). (b) Densitometry analysis of P-AMPK*α* expression (mean ± SEM, ^*^ *P* < 0.05). (c) Flow cytometry panels representing the effect of AMPK*α*2 knockdown on cell cycle arrest induced by 8 mM metformin (Met), compared with control (Ctrl). (d) High glucose treatment is comparable to AMPK*α*2-deficiency. The cells were cultured in 5.5 mM NG, 25 mM D-glucose (HG), for 48 h. For the last 24 h, 8 mM metformin (Met) was added in the indicated conditions. Data expressed as mean + SEM. NS *P* > 0.05; ^**^ *P* < 0.01, compared with Ctrl.

Metformin is a well described activator of AMPK [40]. HEK293 cells express both the *α*1 and *α*2 isoforms of AMPK; however, it is the *α*2 subunit that is primarily involved in AMPK activation induced by reduced ATP synthesis [41]. According to a widely accepted view, the cellular effects of metformin involve the inhibition of the mitochondrial electron transport chain; therefore, it was expected that metformin would exert its effects in an AMPK*α*2-dependent manner [21, 42]. In order to investigate the involvement of AMPK in cell cycle regulation, the alpha2 isoform of AMPK was knocked down in HEK293 cells by a ShRNA-mediated approach. AMPK*α*2 knockdown was validated by measuring phosphorylation of threonine 172 on both isoforms (*α*1, *α*2) of the alpha subunit (Figures 3(a) and 3(b)). In contrast to the nonsilencing condition, in AMPK*α*2 knockdown cells metformin did not induce cell cycle arrest in the G0/G1 phase (Figures 3(c) and 3(d)). AMPK*α*2 deficiency had a comparable effect to high glucose treatment in reversing metformin-induced cell cycle arrest.

### 3.3. High Glucose Has an Opposing Effect on Cell Size Reduction Induced by Metformin

**Figure 4:**
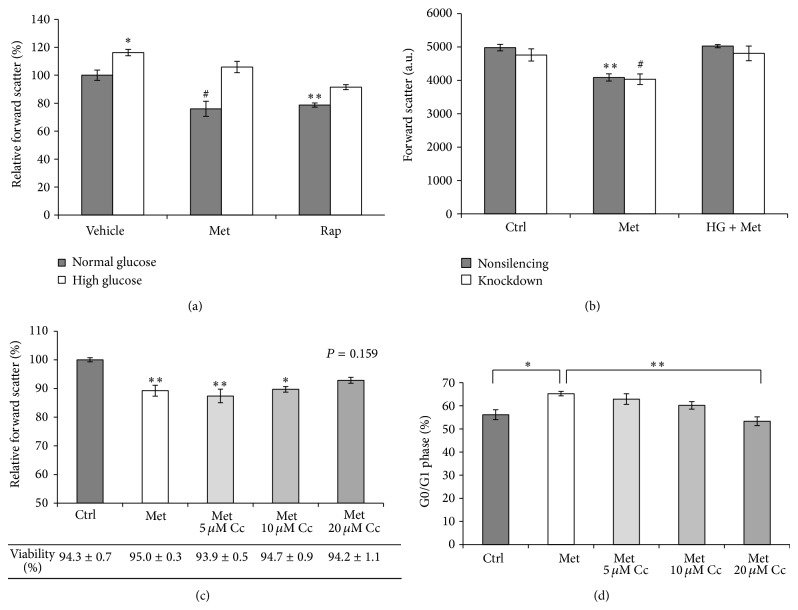
(a) High glucose reverses metformin-induced cell size decrease in HEK293 cells. The cells were cultured with 5.5 mM (Ctrl) or 30 mM D-glucose (HG) for 3 days. For the last 24 h, 8 mM metformin or 100 nM rapamycin was added. Cell size was measured by flow cytometry. (b) AMPK*α*2-deficiency has no effect on metformin-mediated cell size reduction. The cells were incubated in 5.5 or 25 mM (HG) D-glucose for 3 days. For the last 24 h, metformin 8 mM (Met) was added. (c) Compound C reverses metformin-induced cell size reduction. HEK293 cells were cultured with or without (Ctrl) 8 mM Met. At the indicated concentrations, compound C (Cc) was added to the cells and cocultured with Met for 24 h. The viability of the cells was determined by propidium iodide staining. Vehicle effects of compound C were controlled by keeping the concentration of DMSO at 0.2% in the conditions. (d) Dose-dependent reversal of metformin-induced cell cycle arrest by compound C. HEK293 cells were exposed to the experimental conditions described in Figure 4(c). The results are expressed as mean ± SEM of three independent experiments. ^*^ *P* < 0.05; ^**^ *P* < 0.01; ^#^ *P* < 0.001.

Relative to cycling cells, G0/G1 arrested cells have a reduced cell size phenotype [23]. In order to investigate if cell size measurements correlate with the cell cycle experiments, the size of HEK293 cells was measured by flow cytometry. This cell type has been reported to have an intrinsic variation in cell size at various stages of confluence [43]. Nevertheless, in our study no significant differences were detected in the size of HEK293 cells up to 60% confluence level (data not shown). Metformin caused approximately 20% decrease in cell size, which was at a comparable level to the effect of rapamycin (Figure 4(a)). In the combination conditions with high glucose, the effects of the drugs on cell size were reversed, with a more pronounced effect in the metformin-treated condition.

The dependence of metformin on AMPK in cell size regulation was also investigated. In line with the results presented in Figure 4(a), metformin caused a decrease in cell size in the control, nonsilencing condition (Figure 4(b)). In AMPK*α*2-deficient cells metformin inhibited cell size to the same extent as it did in control cells. As expected, high glucose reversed the effect of metformin on cell size in both control and AMPK*α*2-deficient cells. The role of AMPK was further studied by using compound C. Some reports have suggested that compound C may promote cell death through apoptosis [44, 45]. To investigate the toxic effects of compound C, the viability of the cells was determined by propidium iodide staining. Although the viability of cells was not significantly affected by the treatments, cell size was measured on the propidium iodide-negative cell population. Compound C reversed metformin-induced cell size reduction in a concentration-dependent manner, with a maximal effect of compound C at 20 *μ*M (Figure 4(c)). In order to investigate whether the effect of compound C on cell size could be associated with corresponding changes in cell cycle, cell cycle analysis was done on cells exposed to the same experimental conditions described in Figure 4(c). At 20 *μ*M, compound C blunted the effect of metformin on inducing an increase in the G0/G1 phase of the cell cycle (Figure 4(d)).

### 3.4. The Hypertrophic Effects of High Glucose Are Associated with the Activation of the mTOR Signalling Pathway

**Figure 5:**
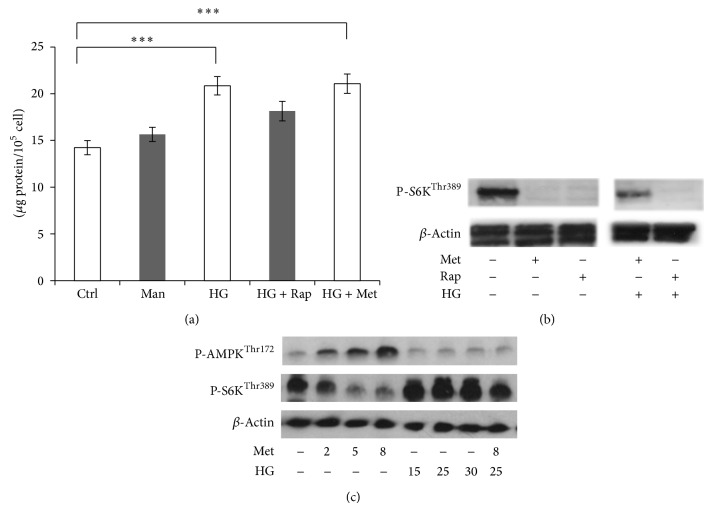
(a) Rapamycin and metformin differentially affect high glucose-induced total protein synthesis. HEK293 cells were incubated with 5.5 mM D-glucose (Ctrl), 5.5 mM D-glucose + 24.5 mM mannitol (Man), and 30 mM D-glucose (HG) for 48 h. For the last 24 h, 100 nM rapamycin (Rap) and 8 mM metformin (Met) were added to HG-treated conditions. The results expressed as mean + SEM of four independent experiments. ^***^ *P* < 0.001. (b) Metformin (Met) and rapamycin (Rap) differentially affect S6K phosphorylation in high glucose pretreated HEK293 cells. The cells were incubated with 5.5 mM (NG) or 30 mM D-glucose (HG) for 3 days. For the last 24 h, 8 mM metformin (Met) or 100 nM rapamycin (Rap) was added. The expression of the indicated proteins was analysed by western blotting. (c) Western blot results of the effect of metformin and high glucose on AMPK and S6K phosphorylation. HEK293 cells were cultured in normal medium supplemented with 5.5 mM D-glucose. At the indicated final concentrations, (mM) metformin (Met) and D-glucose (HG) were added for 24 h and three days, respectively.

An increased cell size can correlate with increased protein content. As an index of high glucose-induced hypertrophy, total protein content was measured in HEK293 cells. The results show that high glucose caused a 25% increase in total protein synthesis over a culture period of two days (Figure 5(a)). This hypertrophic effect of high glucose treatment was reduced by rapamycin. In contrast, metformin did not exert an observable inhibitory effect on total protein synthesis in high glucose-treated cells.

The inhibitory effects of rapamycin and metformin on mTOR signalling were assessed by measuring the phosphorylation level of mTOR and S6K at Ser2448 and Thr389, respectively (Figure 5(b)). Incubating HEK293 cells with 8 mM metformin and 100 nM rapamycin for 24 h reduced mTOR and S6K phosphorylation, with more pronounced effects on S6K. In contrast to the combination condition with rapamycin, 30 mM D-glucose pretreatment had an opposing effect on mTOR inhibition by metformin.

In order to investigate whether high glucose opposes metformin-induced mTOR inhibition via AMPK, as a marker of its activation, we measured the phosphorylation level of threonine 172 on both isoforms (*α*1, *α*2) of the alpha subunit. Metformin activated AMPK in a dose-dependent manner. As expected, AMPK activation resulted in a concomitant decrease in S6K phosphorylation (Figure 5(c)). In the range of 15–30 mM, D-glucose inhibited AMPK phosphorylation and at 25 mM concentration D-glucose treatment opposed the effect of metformin on AMPK/S6K signalling.

### 3.5. AMPK Inhibition Is Associated with the Reversal of Metformin-Induced p21 Downregulation in HEK293 Cells

**Figure 6:**
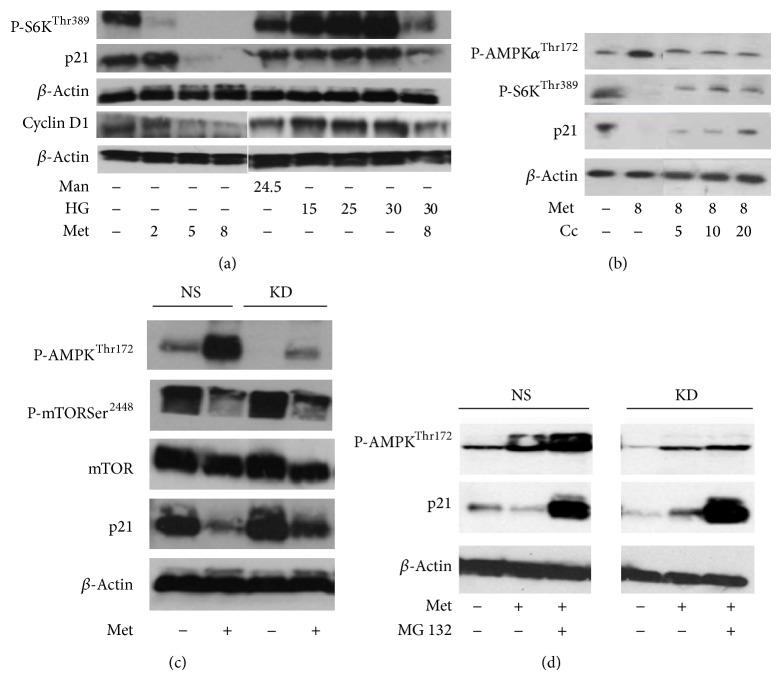
Western blot results indicate that AMPK inhibition is associated with the reversal of metformin-induced p21 downregulation in HEK293 cells. In these experiments, the protein level of P-S6K^Thr389^, cyclin D1, P-AMPK*α*12^Thr172^, and P-mTOR^Ser2448^ was measured in order to confirm the expected effects of treatments. mTOR and *β*-actin were used to control equal protein loading. (a) The cells were treated with culture medium containing 5.5 mM D-glucose. Mannitol (Man) and D-glucose (HG) were added for three days at the indicated concentrations (mM). The cells were treated with metformin (Met) for 24 h at the indicated concentrations (mM). (b) Compound C (Cc) reverses metformin-induced p21 downregulation. The cells were treated with Met and Cc for 24 h at the indicated concentrations (mM and *μ*M, resp.). (c) HEK293 cells stably transfected with shRNA expression plasmids targeting AMPK*α*2 (KD) or nonsilencing (NS) were cultured with or without 8 mM metformin for 18 h in whole cell culture medium. (d) The proteasome inhibitor carbobenzoxy-Leu-Leu-leucinal (MG132) prevents metformin-induced (Met) downregulation of p21. HEK293 cells stably transfected with shRNA expression plasmids targeting AMPK*α*2 (KD) or nonsilencing (NS) were cultured with or without 8 mM metformin and 10 *μ*M MG132 overnight in whole cell culture medium. Vehicle effects were controlled by adding 0.05% DMSO to the conditions.

**Figure 7:**
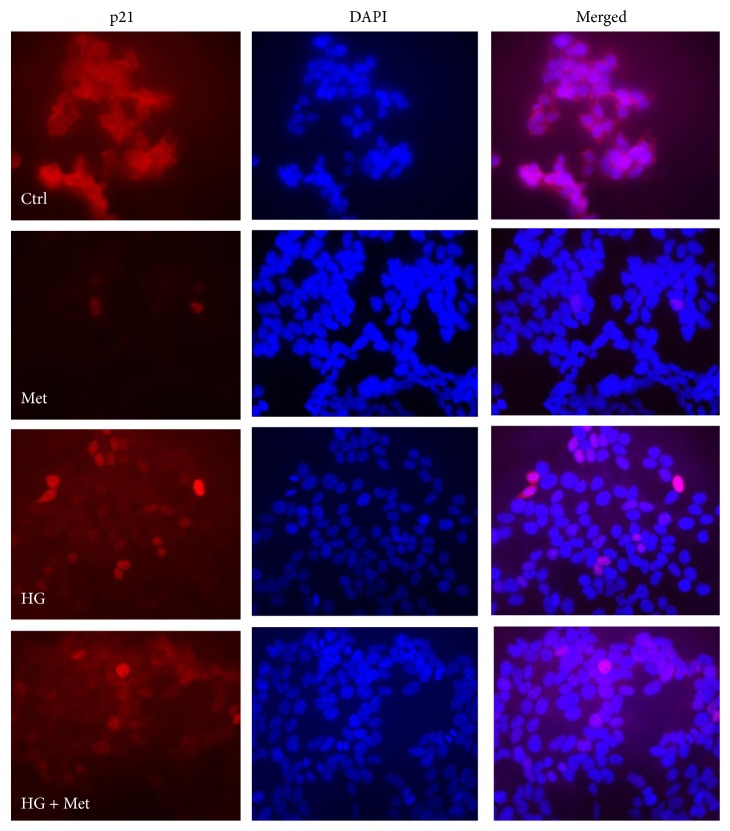
Detection of metformin-induced downregulation of p21 by immunocytochemistry. HEK293 cells were grown on glass slides until 60% confluence. The cells were cultured with 5.5 mM or 25 mM (HG) D-glucose for three days. For the last 24 h, 8 mM metformin (Met) was added to the indicated conditions. Original magnification ×400. Representative image of two independent experiments.

The expression of p21 was investigated in the context of AMPK/mTOR/S6K signalling by western blotting. Metformin treatment caused a decrease in p21 expression in a dose-dependent manner (Figure 6(a)). The expression level of phosphorylated S6K and cyclin D1 was also investigated. As expected, metformin caused downregulation of both P-S6K and cyclin D1 with more pronounced effects in the 5–8 mM concentration range. High glucose treatments (15–30 mM) increased the expression of p21, cyclin D1, and P-S6K and at 25 mM concentration blunted the inhibitory effects of metformin. Similar to high glucose, compound C had an opposing effect of metformin-induced AMPK activation, S6K dephosphorylation, and p21 downregulation (Figure 6(b)). The inhibitory effect of metformin on p21 expression was also confirmed in HEK293 cells stably expressing ShRNA against AMPK*α*2 (Figure 6(c)). In AMPK*α*2-deficient cells, metformin-induced AMPK activation was reduced. Correspondingly, reciprocal changes in mTOR phosphorylation could also be observed. Metformin treatment reduced p21 expression in the nonsilencing control condition. In contrast, in the AMPK*α*2 knockdown condition the inhibitory effect of metformin on p21 expression was less pronounced. The expression of p21 can be regulated by the proteasome and recently it has been suggested that AMPK activation may inhibit the function of the proteasome [46–48]. To investigate whether metformin-induced downregulation of p21 is proteasome-dependent, the proteasome inhibitor, carbobenzoxy-Leu-Leu-leucinal (MG132), was used in control and AMPK*α*2-deficient cells. In both control and knockdown cells, the downregulation of p21 was prevented by MG132 treatments (Figure 6(d)). In order to confirm the above western blot results and obtain information about changes in p21 localisation, immunofluorescence microscopy was performed on metformin and high glucose treated HEK293 cells. As expected, metformin treatment decreased the expression of p21 and high glucose treatment reversed metformin-induced p21 downregulation (Figure 7). In addition, high glucose treatment enhanced the nuclear compartmentalisation of p21.

### 3.6. Inhibition of mTOR Signalling Has no Effect on p21 Expression

**Figure 8:**
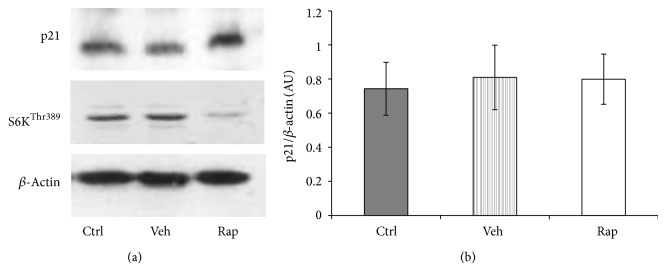
mTOR inhibition by rapamycin does not have a negative effect on p21 regulation. HEK293 cells were incubated with 100 nM rapamycin (Rap) for 24 h. Vehicle effects were controlled by 0.1% ethanol treatments. (a) Representative western blot image of p21 and *β*-actin expression. (b) Band intensity was quantified by densitometry. Data are arbitrary units (AU) expressed as means ± SEM (*n* = 6).

**Figure 9:**
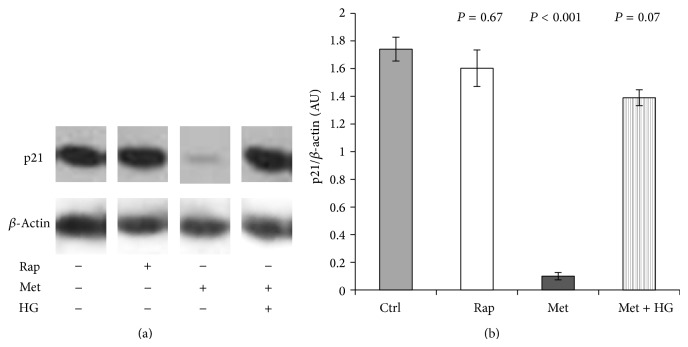
Metformin inhibits p21 expression in podocytes. The cells were treated with 5.5 mM or 25 mM D-glucose for three days. For the last 24 h, 8 mM metformin or 100 nM rapamycin were added. (a) Western blotting was performed for p21 and *β*-actin. (b) Band intensity was quantified by densitometry. Data are arbitrary units (AU) expressed as mean ± SEM (*n* = 3).

mTOR plays a central role in cell cycle regulation [49]. Furthermore, rapamycin downregulates p21 in mouse fibroblasts [50]. In order to investigate whether mTOR inhibition plays a role in p21 regulation, the effect of rapamycin on p21 expression was determined by immunoblotting in HEK293 cells. The inhibition of the mTOR signalling pathway was demonstrated by the reduced level of S6K phosphorylation. Rapamycin did not have an effect on p21 expression (Figure 8). This finding was also confirmed in conditionally immortalised human podocytes that expressed p21 in a metformin-sensitive but rapamycin-insensitive manner (Figure 9).

## 4. Discussion

We have found that in HEK293 cells high glucose opposes the negative effects of metformin and rapamycin on proliferation, cell size, and protein synthesis, parameters that are widely associated with structural changes early in the development of DN [51]. It has also been observed that high glucose differentially affects mTOR-related phenotypes induced by metformin and rapamycin. This differential effect of high glucose may be attributed to inhibition of AMPK activation. Contrary to our expectations, we have found that metformin inhibits p21 expression in a concentration-dependent manner, independently of its effect on mTOR signalling. High glucose, AMPK*α*2-deficiency, and compound C overcome the inhibitory effect of metformin on p21 expression, suggesting that AMPK may play a role in the regulation of this cyclin-dependent kinase inhibitor.

Early cellular changes in the development of DN involve hyperplasia and hypertrophy in both the tubular and mesangial compartments [4]. These cellular changes have been attributed to increased cellular glucose uptake in cells that are not protected from high ambient glucose levels [52, 53]. In the past decades a considerable interest has been devoted to cell cycle regulatory proteins in glomerular hypertrophy [54–57]. These studies have suggested that the initial pathological changes in the diabetic kidney are associated with low-grade proliferation followed by cellular hypertrophy.* In vitro* and* in vivo* studies have shown that the absence of p21 prevents the development of hypertrophy associated with the diabetic kidney [56, 58]. It has been shown that the expression of p21 gene (*CDKN1A*) is robustly induced in both type 1 and type 2 diabetic animals [7]. According to a widely held view, high p21 expression is associated with cell cycle arrest [59–61]. However, the role of p21 as a cell cycle inhibitor has been challenged by the finding that p21 may serve as an assembly factor for cyclin D-Cdk4 complex formation. The cyclin D-Cdk4 complex is required for cell cycle progression and is known to be ubiquitous among different cell types [62, 63]. Furthermore, several studies have suggested that increased cyclin D1 expression is associated with renal and cardiac hypertrophy, which may be attributed to increased stabilisation of p21 [64–66]. In this study, high glucose opposed the inhibitory effects of metformin on both cyclin D1 and p21 expression. In addition, these effects of high glucose correlated with the promotion of cell cycle progression in metformin treated cells, suggesting that high glucose-induced expression of cyclin D1 and p21 may be linked by a common mechanism.

The results of this study suggest that inhibition of AMPK may be the underlying mechanism by which high glucose induces p21 expression, which in turn may stimulate proliferation and cell growth. The involvement of AMPK in the regulation of proliferation has been reported in other studies [26, 27, 67]. In contrast to the *α*1 isoform, AMPK*α*2 has been shown to respond to stress-related conditions, such as hypoxia and glucose deprivation. Furthermore, in AMPK*α*2 knockout mice the antihypertrophic effects of metformin are known to be attenuated [68]. These findings suggest that energy-depleting agents, such as metformin or AICAR, exert their antihypertrophic effects through activation of this isoform [69]. However, both drugs may have some limitations in the interpretation of their cellular actions. It has been reported that, in a time- and concentration-dependent manner, AICAR may act as an ATP analogue due to the increase in ZTP, its triply phosphorylated form [70]. Furthermore, it has been suggested that AICAR may also mediate AMPK-independent processes by regulating AMP-sensitive enzymes, such as glycogen phosphorylase [71]. AMP-independent activation of AMPK by metformin has also been reported [72, 73]. However, a recent study has demonstrated that AICAR-induced AMPK activation downregulates p21 expression in retinoblastoma cells [74]. Similarly, despite inhibition of cell cycling, AICAR treatment reduced p21 levels in myoblast cultures [75]. The results of this latter study also show that inhibition of AMPK with compound C associates with the reversal of AICAR-induced loss of p21 expression. Our finding that compound C abrogates metformin-induced cell cycle arrest is in line with the concept that AMPK inhibition promotes proliferation. Furthermore, in experiments studying metformin-induced cellular mechanisms, compound C has been successfully used for blunting AMPK activation [76, 77]. In accordance with these studies, our results also show that compound C opposes the effects of metformin on the AMPK/S6K pathway and p21 expression. These latter findings suggest that AMPK inhibition may promote proliferation through increased p21 expression.

The effects of metformin have generally been associated with the activation of AMPK [21]. Evidence suggests that metformin may lower the risk of cancer in patients with diabetes [78]. In cancer studies the antiproliferative effect of metformin has been attributed to its ability to induce cell cycle arrest through an AMPK-dependent mechanism [17, 27, 29]. According to a suggested mechanism, through the activation of AMPK, metformin downregulates cyclin D1, leading to the release of sequestered CDK inhibitors, p27 and p21. In turn p27 and p21 may associate with E/CDK2 complexes and inhibit cell cycling at the G1/S checkpoint [29]. On the other hand, in melanoma cells p21 expression was not required for metformin-induced cell cycle arrest [79]. Our results show that both metformin and rapamycin inhibit proliferation in HEK293 cells by inducing cell cycle arrest in the G0/G1 phase. Interestingly, high glucose pretreatment abrogated the inhibitory effect of metformin on cell cycling but did not affect rapamycin-induced cell cycle inhibition. Furthermore, rapamycin neither increased nor decreased p21 expression in this study. These findings suggest that increased p21 expression is not required for cell cycle inhibition by metformin and rapamycin.

The involvement of cell cycle regulatory proteins in the development of DN has long been suggested [5, 56, 80–82]. These studies have suggested that glomerular hypertrophy is a consequence of increased cyclin-dependent kinase inhibitor expression-mediated cell cycle arrest. However, that role of p21 in pathogenesis is more complex. The expression of p21 is generally associated with regulation of cell cycle, apoptosis, and ameliorating DNA damage [83]. In relation to the AMPK/mTOR signalling pathway, the induction of p21 as a senescence marker has been described with relevance to age-related diseases [84].

Accumulating evidence indicates that senescence may play an important role in the development of DN. Premature senescence has been observed in fibroblasts and proximal tubular cells isolated from patients with DN [85–87]. Downregulation of connexin 43, a gap junction protein, has been reported in podocytes of diabetic patients as well as in high glucose treated glomerular mesangial cells that showed increased expression of senescence markers, such as p21, p27, and *β*-galactosidase staining [82, 88]. Connexin 43 has also been implicated in glomerular hypertrophy [89]. Furthermore, high glucose induces cyclin D1 expression, and interestingly, increased cyclin D1 expression is associated with senescence in fibroblasts [90]. Diabetes contributes to vascular ageing, and endothelial cell senescence is induced by high glucose or advanced glycosylated end products [91]. In a study on rat kidney proximal tubule cells, an increase in the expression of p21 and p27 was associated with a phenotypic transition to senescence [8]. Differentiated cells cannot progress to senescence. However, it has been shown that dedifferentiation of cells in the kidney may contribute significantly to the pool of proliferating cells [92, 93]. In turn, dedifferentiated cells responding to mitogenic factors can progress to senescent arrest [59]. Overexpression of fibronectin, one of the extracellular matrix proteins induced in diabetic nephropathy, is one of the characteristics of senescence cells [94]. Furthermore, proteasomal protein degradation is reduced in senescent cells, which can be a contributing factor for increased hypertrophy [95].

The involvement of apoptosis in diabetic nephropathy has been indicated by several investigators [96–98]. It has been widely acknowledged that apart from senescence, damaged cells rely on apoptosis to evade tumour formation [99]. It has been suggested that apoptotic cell loss may be a process of normal tissue homeostasis regulating mesangial cell populations in enlarged glomeruli [100, 101]. Jung et al. [96] have demonstrated using diabetic rats that apoptosis may differentially affect glomerular cells in small and large glomeruli. They also showed that cellular hypertrophy was responsible for the differences in size, as the expression level of fibronectin, an extracellular matrix marker, was the same in both small and large glomeruli. Increased expression of proapoptotic proteins was found in large glomeruli, suggesting that apoptosis may selectively operate in a more hypertrophied cellular environment. Interestingly, upregulation of cyclin D1, p21, and p27 was only detected in smaller glomeruli, indicating that the higher expression of these proteins is associated with the initiation of glomerular hypertrophy.

Inhibition of mTOR by rapamycin has been found to prevent the permanent loss of proliferative potential that is characteristic of cellular senescence [102]. Since metformin also inhibits the mTOR signalling pathway, a similar effect of metformin to that of rapamycin could be expected in the prevention of senescent transformation. However, in contrast to rapamycin, chronic exposure to lower doses of metformin has been shown to promote cancer-protective senescence in other studies [40, 103]. It has been proposed that rapamycin regulates proliferation by preventing the formation of cyclin-dependent kinase complexes. mTOR activation by growth factors or high energy levels induces p21 that in turn facilitates the assembly of these complexes [104]. In this study metformin inhibited p21 in both HEK293 cells and human podocytes, suggesting that energy depletion may affect the expression of this cell cycle inhibitor. The underlying factor that determines the cellular actions of AMPK may be its level of activation [84, 105]. However, a major limitation to understanding the role of AMPK in the diabetic kidney is the lack of studies investigating cell-specific differences in AMPK expression [106]. Cells in the medulla predominantly depend on glycolytic metabolism, whereas tubular cells in the cortex depend on oxidative metabolism [107, 108]. Glycolytic flux is known to be reduced during the process of cellular senescence; therefore one can envisage that senescence-induced metabolic disturbances may differentially impact on cell populations in the kidney [109, 110]. Immortalised cells may have a relatively high glycolytic flux; therefore, it is possible that in our experiments high glucose exerted its effects through enhanced glycolysis [111].

In preclinical studies, the doses of metformin are much higher than the level of the drug reported to accumulate in tissues after oral administration; therefore, it is difficult to extrapolate the results of these studies to a clinical setting because of the differential and possibly poor expression of OCTs in immortalised cell lines [21]. Nevertheless, these studies show that metformin may have beneficial effects in pathologic states, including cancer and inflammation [17, 112, 113]. Several molecular pathways in these diseases are also relevant to the aetiology of diabetic nephropathy, suggesting that metformin may have a therapeutic potential for the prevention of DN. Indeed, by modulating the expression of proinflammatory genes, metformin has been shown to ameliorate DN in rats and AMPK activation by metformin reduced renal hypertrophy in diabetic rats [114, 115].

## 5. Conclusion

In summary, the above indicates that metformin can reduce the expression of p21 and AMPK may play a role in the underlying mechanism. It can also be inferred from these results that p21 is not required for metformin-induced cell cycle arrest. This finding lends support to other studies that look beyond the role of p21 as a cell cycle inhibitor [74, 75]. Metformin has implications for the treatment of both diabetes and cancer and p21 has different, poorly understood roles in both diseases. Recently, energy sensing pathways have been investigated in the context of both diabetic complications and cancer [116–118]. In addition, reentry of differentiated cells into the cell cycle as well as genetic polymorphism in the p21 gene has been implicated in Alzheimer's disease [119, 120]. Our findings may prompt further research in these fields.
